# Impact of forest landscape restoration in combating soil erosion in the Lake Abaya catchment, Southern Ethiopia

**DOI:** 10.1007/s10661-024-12378-8

**Published:** 2024-02-02

**Authors:** Shibire Bekele Eshetu, Harison Kiplagat Kipkulei, Julian Koepke, Harald Kächele, Stefan Sieber, Katharina Löhr

**Affiliations:** 1 Leibniz Centre for Agricultural Landscape Research (ZALF), Eberswalder Str. 84, 15374 Müncheberg, Germany; 2 Thaer-Institute for Agricultural and Horticultural Sciences Agricultural Economics, Humboldt Universität zu Berlin, Invalidenstr, 42, 10115 Berlin, Germany; 3 Department of Geomatic Engineering and Geospatial Information Systems, Jomo Kenyatta University of Agriculture and Technology (JKUAT), Nairobi, Kenya; 4 Eberswalde University for Sustainable Development, Schicklerstraße 5, 16225 Eberswalde, Germany; 5 Urban Plant Ecophysiology, Thaer-Institute for Agricultural and Horticultural Sciences, Humboldt Universität zu Berlin, Lentzeallee 55/57, 14195 Berlin, Germany

**Keywords:** RUSLE, GIS, Erosion, Landscape restoration, Scenario analysis

## Abstract

As an effect of forest degradation, soil erosion is among Ethiopia’s most pressing environmental challenges and a major threat to food security where it could potentially compromise the ecosystem functions and services. As the effects of soil erosion intensify, the landscape’s capacity to support ecosystem functions and services is compromised. Exploring the ecological implications of soil erosion is crucial. This study investigated the soil loss and land degradation in the Lake Abaya catchment to explore forest landscape restoration (FLR) implementation as a possible countermeasure to the effects. The study used a geographic information system (GIS)-based approach of the Revised Universal Soil Loss Equation (RUSLE) to determine the potential annual soil loss and develop an erosion risk map. Results show that 13% of the catchment, which accounts for approximately 110,000 ha, is under high erosion risk of exceeding the average annual tolerable soil loss of 10 t/ha/year. Allocation of land on steep slopes to crop production is the major reason for the calculated high erosion risk in the catchment. A scenario-based analysis was implemented following the slope-based land-use allocation proposal indicated in the Rural Land Use Proclamation 456/2005 of Ethiopia. The scenario analysis resulted in a reversal erosion effect whereby an estimated 3000 t/ha/year of soil loss in the catchment. Thus, FLR activities hold great potential for minimizing soil loss and contributing to supporting functioning and providing ecosystem services. Tree-based agroforestry systems are among the key FLR measures championed in highly degraded landscapes in Ethiopia. This study helps policymakers and FLR implementors identify erosion risk areas for future FLR activities. Thereby, it contributes to achieving the country’s restoration commitment.

**Supplementary Information:**

The online version contains supplementary material available at 10.1007/s10661-024-12378-8.

## Introduction

As the population continues to increase, land degradation is amplified, leading to a decline in crop productivity (Mitiku et al., 2006). In particular, the deforestation of mountain forests and landscape use of large areas leads to severe soil degradation through erosion processes (Reusing et al., 2000). Soil erosion is reported to be a threat to agroecosystems and one of the main global environmental problems (Bayramin et al., 2003; Blanco & Lal, 2008; Montanarella et al., 2016; Pimentel, 2006). Resource loss due to land degradation by soil erosion is a problem globally and is found to be particularly high in East African countries (Kirui & Mirzabaev, 2014). Ethiopia bears an annual cost of USD 700 million due to nutrient loss by water erosion from croplands (Hurni et al., 2015). For human use, many areas of the Ethiopian highlands have been deforested for firewood and timber, and the land is used for cropping and grazing. These anthropogenic influences lead to damage to the soil so that, among other things, large parts of the highlands are very vulnerable to soil erosion (Pistorius et al., 2017; Reusing et al., 2000).

The impacts of climate change further intensify the problem of soil degradation in the region (Pistorius et al., 2017), leading to major challenges in ensuring food security for Ethiopia’s current and future population (Mitiku et al., 2006). Land degradation is to be prevented to secure agricultural land and, if possible, increase its productivity (Hurni et al., 2015). According to Stanturf et al. (2019), FLR aims to regain ecological functionality and contributes to building a resilient ecosystem in response to climate change which enhances agricultural productivity. FLR options such as agroforestry practices can be an intervention to reduce soil loss resulting from the expansion of agricultural land.

The expansion of agricultural activities at the expense of the trailing forest areas in the Ethiopian highlands and the Rift Valley is exerting pressure on natural resources (Ayenew & Legesse, 2007; Meshesha et al., 2012). Even though expanding agricultural land increases the coverage under agricultural production, it poses a danger of soil erosion and does not necessarily lead to improved production (Ayenew & Legesse, 2007). The Ethiopian Rift Valley area is among the high erosion–risk areas with poor agricultural productivity (Meshesha et al., 2012). Deforestation and the resulting land degradation have also affected the quality and quantity of water resources in the region. For example, Lake Abaya, the largest Rift Valley lake separated by a 5-km-wide ridge with an elevation difference of 60 m from Lake Chamo, has experienced a higher concentration of suspended solids and sediment accumulation as compared to Lake Chamo (Awulachew, 2006; Teffera et al., 2017; Teffera et al., 2019). This has been caused by increased deforestation, conversion of natural forest to cropland, and land degradation in the main catchment (Meshesha et al., 2012; Teffera et al., 2017). Hence, the tributaries load significant sediment from the catchment to Lake Abaya (Teffera et al., 2019). To minimize the risk, estimating the amount of soil loss in the catchment and identifying erosion risk areas to implement site-specific FLR options is crucial.

To understand the soil loss dynamics, the Universal Soil Loss Equation (USLE) was developed in 1978 which predicts the long-term average annual rate of erosion on field slopes based on rainfall pattern, soil type, topography, crop system, and management practices. The equation was further improved, and the successor Revised Universal Soil Loss Equation (RUSLE) was developed, which is land-use independent (Renard, 1997). Compared to USLE, the revised equation robustly captures all land uses and better calculations for the slope length and steepness (*LS*) factor. Furthermore, the revised version is more advanced in computerization to determine individual factors (Jain & Singh, 2003).

The RUSLE model has a long history of estimating soil loss by analyzing major variables of soil erosion by water (Renard, 1997). Even though the RUSLE model is a convenient method for estimating erosion for river basins and individual farm fields due to sheet and rill erosion types, deterioration of erosion by land sliding and gully is not captured by this model (Jain et al., 2001; Teng et al., 2018). However, direct soil loss measurements using classical erosion plots are costly and time-consuming, especially over large areas. Hence, the RUSLE model is the best alternative soil erosion model that can be implemented in remote sensing (RS) and geographic information system (GIS) environments to estimate potential long-term average annual soil loss (Lee & Lee, 2006; Van Remortel et al., 2001). Integrating RS and GIS in the RUSLE model enables the estimation of soil loss and its spatial distribution at a lower cost and greater accuracy across large areas (Alexakis et al., 2013; Rahman et al., 2009). GIS and RS analysis can generate catchment-scale soil erosion risk maps. These maps provide useful insights into the linkage between soil erosion and sediment deposition in a given catchment (Blinkov & Kostadinov, 2010). Thus, it helps to develop sound management strategies and prioritization of watershed management intervention at catchment scales (Ayele et al., 2017; Ganasri & Ramesh, 2016). The overall objective of this study is to estimate the annual soil loss and to generate an erosion risk map of the Abaya catchment. Specifically, the study computes the factors of erosion, identifies erosion risk distribution by location, and simulates how potential FLR options can minimize the erosion hotspot areas in the catchment.

## Methodology

### Study area

**Fig. 1:**
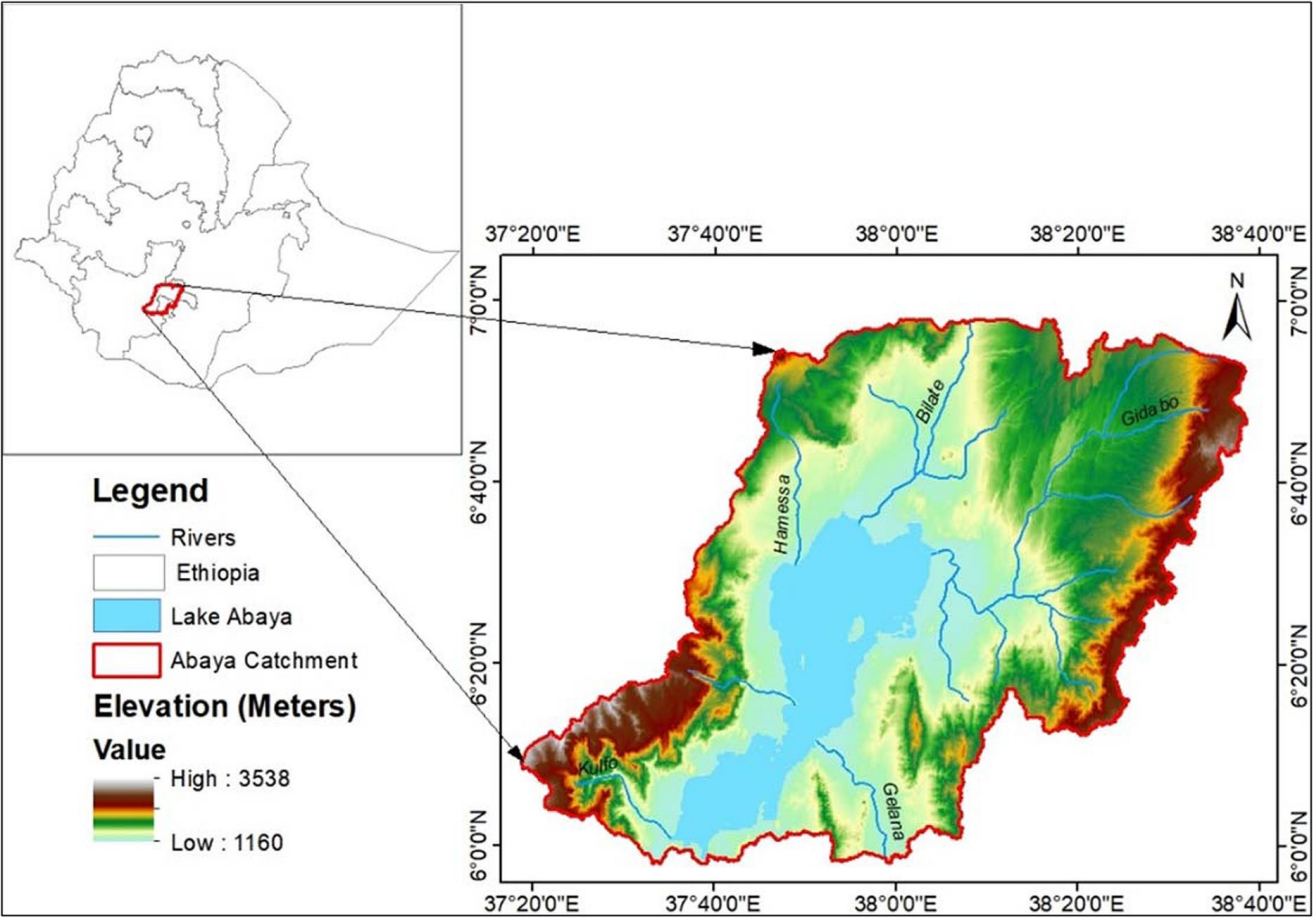
Study area map (source: own map)

The study was conducted in the Lake Abaya catchment situated in the Rift Valley of southern Ethiopia. The Lake Abaya catchment covers an area of 1.86 million ha, including the Lake area. It is mainly fed by major drainage systems of the Bilate River from the north, Gidabo and Galena from the east, and Hare, Hamessa, and Baso from the west (Awulachew, 2006). The southern part of the catchment is connected to Lake Chamo, whereby drainage is possible from Lake Abaya to Chamo in cases of overflow. Due to the silty and clayey sediment transported by the tributaries, the water of Lake Abaya is reported to be reddish-brown (Schütt et al., 2002). The major tributary of the catchment is the Bilate River draining about 5754 km^2^. Figure 1 shows the location of the study area and the rivers draining to Lake Abaya.

### Data type and data source

Both primary and secondary data have been used to estimate the annual soil loss in the area. Secondary data on average monthly rainfall data for the last 30 years (1991 to 2021) was obtained from the National Meteorological Agency of Ethiopia (NMA). Slope, soil, and land-use information were obtained from the US Geological Survey (USGS), iSDA, and Environmental Systems Research Institute (ESRI), respectively. The Shuttle Radar Topographic Mission (SRTM) digital elevation model (DEM) from USGS was used to derive the slope *LS*-factor clipped to the study area extents. Soil and water conservation measures in the area as the land management factor were triangulated with Google Earth Maps, a literature survey, and field observations.

The iSDA soil maps provided soil fractions used to estimate the soil erodibility (*K*) factor. The iSDA soil layer is a new soil map service for the whole African continent provided at a spatial resolution of 30 m. The layer was first introduced in 2021, and according to the authors, the dataset is the first soil map service with a finer resolution at a continent scale worldwide (Hengl et al., 2021). The iSDA soil layer was built on the African Soil Information Service (AfSIS) and the Africa Soil Profile Database (AfSP) projects, both by the International Soil Reference and Information Centre (ISRIC). The soil profiles collected in the projects were combined with other datasets, standardized, harmonized, (statistically) processed, and generalized to large scales using machine learning approaches (iSDA, 2022). The clay, silt, and sand fractions are mapped as mass percentages, and the soil organic carbon (SOC) is represented in grams per kilogram of soil mass in the dataset.

A land-use map from ESRI was used to extract the land cover (*C*) factor. ESRI developed a standardized global land-use map with a fine resolution of 10 m, based on the European Space Agency (ESA) Sentinel-2 images. The map provides land cover information for 2020, and categorizes land into nine different land-use/cover classes, including clouds, with no derived land cover information. ESRI stated an overall classification accuracy of 86%. The soil conservation factor also the land management, *P*-factor, is intended to consider the practices used on agricultural land to reduce soil loss through surface runoff (Wischmeier & Smith, 1978). The factor, derived from related studies, resulted primarily from slope classes derived from the DEM.

### Data analysis

#### Soil loss analysis

RUSLE equation was applied to calculate the annual soil loss from the study area and possible erosion mitigation through applying simulated FLR in the erosion risk area. The steps followed to estimate the annual soil loss of the area using the RUSLE equation are elucidated as follows.

1 $$A=R\times K\times LS\times C\times P$$

where

*A*: is the computed amount of the average annual soil loss per unit area (t/ha^−1^/year^−1^)
*R*: is the rainfall erosivity (MJ mm/ha^−1^/h^−1^/year^−1^)
*K*: is the soil erodibility (t ha/h/ha^−1^/MJ^−1^ mm^−1^)
*LS*: is the slope length and slope steepness factor (dimensionless)
*C*: is the ground cover management (dimensionless)
*P*: is the conservation practice (dimensionless)

The equation gives the soil loss in t/ha/year. The calculation and estimation of various factors are explained below.

##### *R*-factor

The rainfall erosivity factor reflects the influence of precipitation on soil loss. Following Hurni (1985) and Reusing et al. (2000), the 𝑅-factor was calculated using the following formula:

2 $$R=-8.12\times 0.562P$$

In the formula, 𝑃 is the mean annual rainfall in millimeters (Reusing et al., 2000), following the determination of long-term mean annual precipitation for this study, 30 years. The *R*-Factor for the meteorological stations in the study area was calculated and mapped using the geostatistical package in the ArcGIS software version 10.8.2.

The annual mean precipitation from weather stations in the study area ranging between 5.9 to 6.1° N and 37.3 to 38.6° E is used to calculate the *R*-factor values. Table 1 shows the annual mean precipitation from January 1991 to December 2020 in the covered meteorological stations and the calculated *R*-factor values.

**Table 1:** Thirty years of mean precipitation for the NMA stations with the resulting *R* values

Name of weather station	Latitude (°)	Longitude (°)	Average precipitation	Calculated *R*-factor
Arbaminch	6	37.6	936	517
Gedeb	5.9	38.2	1478	823
Gerese	5.8	37.3	2156	1204
Haisawita	6.9	38.6	1128	626
Humbo Tebela	6.7	37.8	1119	621

##### *K*-factor

The resistance of the soil against erosion as a result of rain interception and the rate and amount of runoff produced for the rainfall impact usually depends on geological and soil features (Hadas, 1994; Ugese et al., 2022). The equation by Wawer et al. (2005) was applied with data on soil mass as a percentage of sand, silt, clay, and organic carbon for this study.

3 $$K_\text{USLE}=K_w=f_{c\text{sand}}\;\times\;f_{\text{cl}-\text{si}}\times f_\text{orgc}\times f_{\text{hisand}}$$

where the meaning of the factors is derived as follows: *f*_*c*-sand_ corrects *K*_USLE_ downwards in soils with a high coarse sand content and upwards in soils with low sand content. *f*_cl-si_ corrects *K*_USLE_ downwards in soils with a high clay-to-silt ratio.

4 $${f}_{c-\mathrm{sand}}=\left(0.2+0.3\times \exp \left[-0.256\times {m}_s\times \left(1-\frac{m_{\mathrm{silt}}}{100}\right)\right]\right)$$

5 $${f}_{\mathrm{cl}-\mathrm{si}}={\left(\frac{m_{\mathrm{silt}}}{m_c+{m}_{\mathrm{silt}}}\right)}^{0.3}$$

*f*_orgc_ corrects *K*_USLE_ downwards in soils with high organic carbon content.

6 $${f}_{\mathrm{org}\mathrm{c}}=\left(1-\frac{0.25\times \mathrm{org}C}{\mathrm{org}C+\exp \left[3.72-2.95\times \mathrm{org}C\right]}\right)$$

*f*_hi-sand_ corrects *K*_USLE_ downwards in soils with extremely high sand content.

7 $${f}_{\mathrm{hi}-\mathrm{sand}}=\left(1-\frac{0.7\times \Big(1-\frac{m_s}{100}}{1-\frac{m_s}{100}+\exp \left[-5.51+22.9\times \left(1-\frac{m_s}{100}\right)\right]}\right)$$

whereby the following applies

*m*_*s*_: is the mass percentage of sand
*m*_silt_: is the mass percentage of silt
*m*_c_: is the mass percentage of clay
org*C*: is the mass percentage of organic carbon

##### *LS*-factor

The *L*-factor premises in the slope lengths, while the *S*-factor considers the slope steepness (Wischmeier & Smith, 1978). This combined *LS*-factor is also referred to as the topography, Factor (Amsalu & Mengaw, 2014). Using flow accumulation, slope length was identified, and finally, the *LS*-factor was calculated using Eq. 8 following Stone and Hilborn (2012).

8 $$LS=\left[0.065+0.0456\left(\mathrm{slope}\right)+0.006541\left(\mathrm{slope}\right)2\right]\times \left(\mathrm{slope}\ \mathrm{length}\div 22.1\right)0.5$$

where 22.1 is a metric constant, slope means the slope steepness in percentage, slope length is the length of slope in meters (m), and 0.5 is a potency that applies if slopes ≥ 5 (the case in bigger mountainous regions). The capability of USLE to extract slope length and steepness at a regional level is limited, which is addressed by the above approach (Van Remortel et al., 2004). It generally applies that the longer and steeper the slopes, the higher the erosion risk (Stone & Hilborn, 2012).

##### *C*-factor

The cover management factor reflects the capability of the vegetation covering the soil to prevent soil loss. It is represented by *C* values for the land cover depending on the vegetation type. The basis for the area-wide determination of the *C*-factor is the ESRI 2020 land-use classification map. The determined values were assigned to the correct land-use classes as illustrated in Table 2 and formatted to raster data.

**Table 2:** Values for land-use classes in the study area after various authors

ESRI land-use classes	*C* values	Reference
Water, clouds	0	Gelagay and Minale (2016)
Trees	0.01	Hurni (1985), Gelagay and Minale (2016), Tiruneh and Ayalew (2015)
Grass	0.05	Tiruneh and Ayalew (2015), Degife et al. (2021), Bekele and Gemi (2021)
Flooded vegetation	0.35	Tiruneh and Ayalew (2015)
Crops	0.15	Hurni (1985), Moges and Bhat (2017), Bekele and Gemi (2021), Degife et al. (2021)
Scrub/shrub (not dense or shrubs with grasses)	0.2	Tiruneh and Ayalew (2015), Degife et al. (2021)
Built area	0.15	Hurni (1985), Anteneh and Biru (2021)
Bare ground	1	Hurni (1985), Wischmeier and Smith (1978)

##### *P*-factor

The set *P*-factor is dependent on anthropogenic land use incorporating designated soil conservation measures that are classified based on the slope of the terrain. The land use, therefore, has to be reclassified after that criterion. To set the actual soil conservation values for these reclassified areas, knowledge of the soil conservation methods used on site, as exemplified above, is required. Field observation was conducted on the type of land management soil and water conservation structures in the study area. Constructed stone and soil bunds, fanya juu terraces are the most common land management practices found on the steep agricultural landscape. The field observation was harmonized with the existing *P*-factor values developed for the adjacent catchment, Lake Chamo, which shares the same basin within the Ethiopian Rift Valley (WoldeGabriel et al., 2000). Thus, the *P*-Factor calculation was estimated through combined literature findings from Wischmeier and Smith (1978) and Molla and Sisheber (2017).

At the final stage of the calculated soil erosion factors, the RUSLE equation was applied, and the erosion risk map for the catchment was developed.

#### Scenario analysis

In the scenario analysis, we adopted the Rural Land Proclamation 456/2005 of the Federal Democratic Republic of Ethiopia declares that land situated on greater than 60% slopes can not be used for farming and grazing. The proclamation further declares that rural land areas within a slope class of 30 to 60% can only be used for cropping if bench terraces are constructed. Hence, the proposed land use for the current land area above 60% is a potential land area for the implementation of FLR, which would modify the *C* value and contribute to a potential reduction of the annual soil loss. In the scenario of area-wide FLR implementation, the new *C*-factor value takes the *C* value of the forest. To complement the physical soil erosion measures for the cropland situated within a slope range of 30 to 60%, we adapt a scenario of integrating agroforestry practice. Thereby, croplands situated in this slope range are simulated for agroforestry practice and assigned a *C*-factor value between cropland and forest which is 0.08. The bareland under current land-use classes was all assigned for area-wide FLR measures, with the *C* values of forest in the scenario analysis. The scenario analysis procedure with the old and new *C*-factor values is indicated in Table 3. We finally developed an erosion risk map with simulated FLR options as a strategy to minimize the soil loss from the catchment.

**Table 3:** Proposed changes in land use for intended scenario of FLR impact on soil erosion

Current land use/land cover	Slope class in (Yang et al.)	Proposed land use/land cover	Old *C*-factor value	New *C*-factor value
Bareland	Any range	Forest	1	0.01
Cropland	0–30	Crop	0.15	0.15
Cropland	30–60	Tree-based cropland (agroforestry)	0.15	0.08
Cropland	> 60	Forest	0.15	0.01

## Results

### Annual soil loss factors

#### Rainfall erosivity (R-factor)

**Fig. 2:**
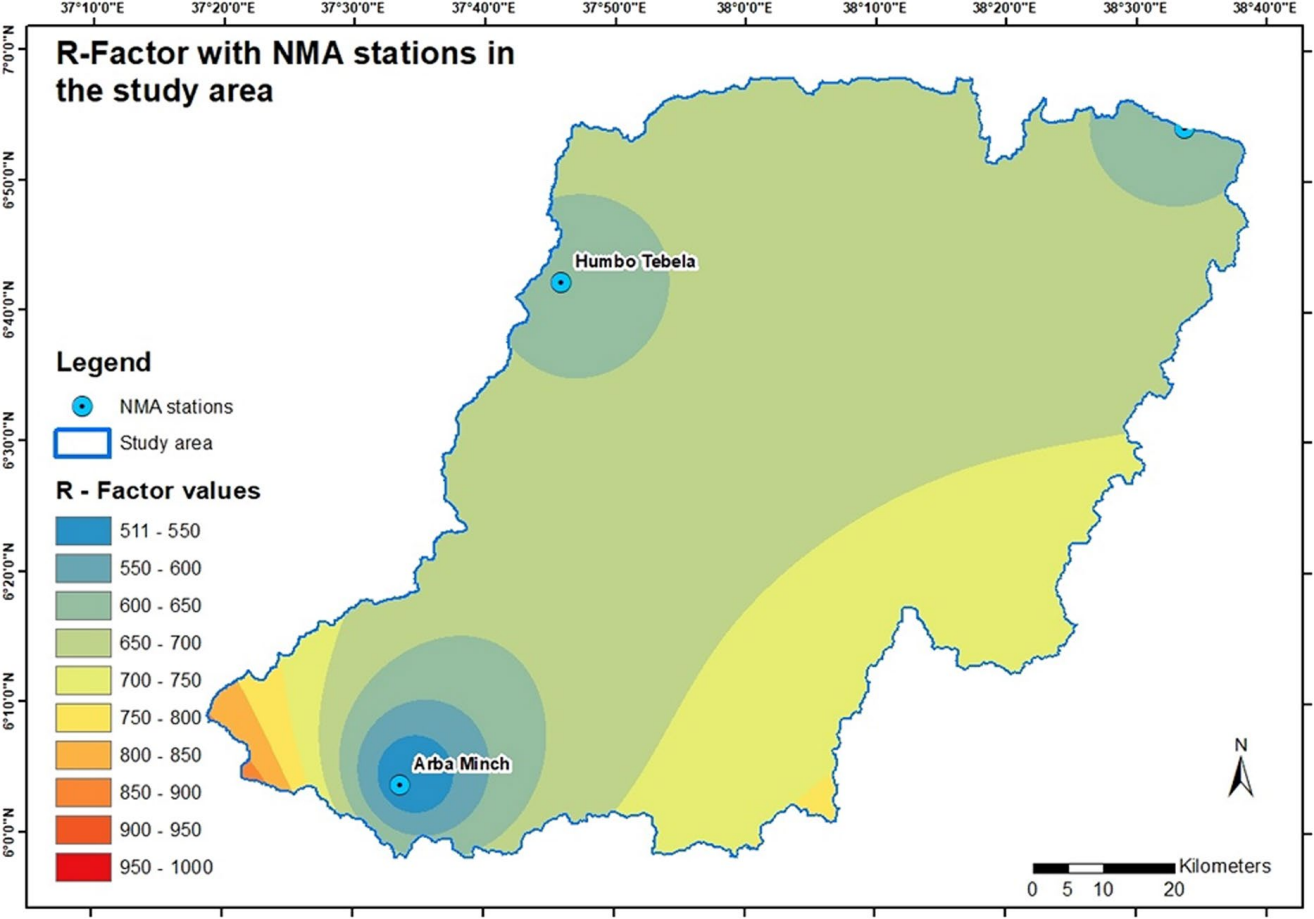
*R*-factor in the study area with the NMA weather stations within the catchment

The results show the area-wide *R* value ranges as *R*-factor based on the measured data points. The highest values cover a small portion of the study area in the extreme southwest, while the lowest values fall within Arbaminch City in the southern part of the study area. The *R* values in the southeast are also high but decline toward the central region. Low *R* values are also found in the northeastern and northwestern parts of the study area. The central and intermediate areas are in the middle range. Figure 2 shows the resulting layer for the area-wide *R*-factor.

#### Soil erodibility (K-factor)

**Fig. 3:**
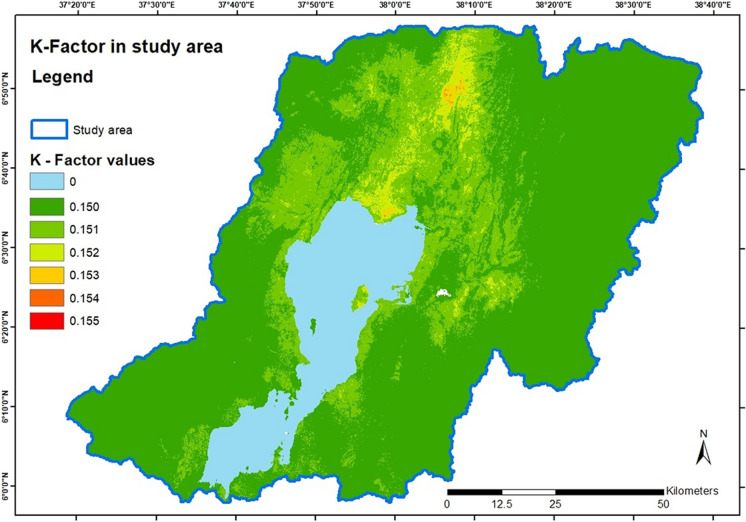
*K*-factor values distribution over the study area

The results from sub-equations for *f*_cl-si_ and *f*_hi-sand_ yielded a value of one over the entire area and thus did not influence the overall equation. The sub-equation for *f*_*c*-sand_ shows some areas of higher values are spread around Lake Abaya. In comparison, *f*_orgc_ produces higher values in the central northern and central-eastern as well as on the northern coast and the largest island of Lake Abaya. Figure 3 shows the resulting spatial distribution of the *K*-factor values. The results show a variance of 0.005 between the lowest and highest *K* values. There are also some clusters of high *K* values within this range of variance, in the north, northern center, and eastern center, with the north showing the highest clustering. The highest individual values are in the north and the northern center.

#### Slope length and steepness: topographic (LS-factor)

**Fig. 4:**
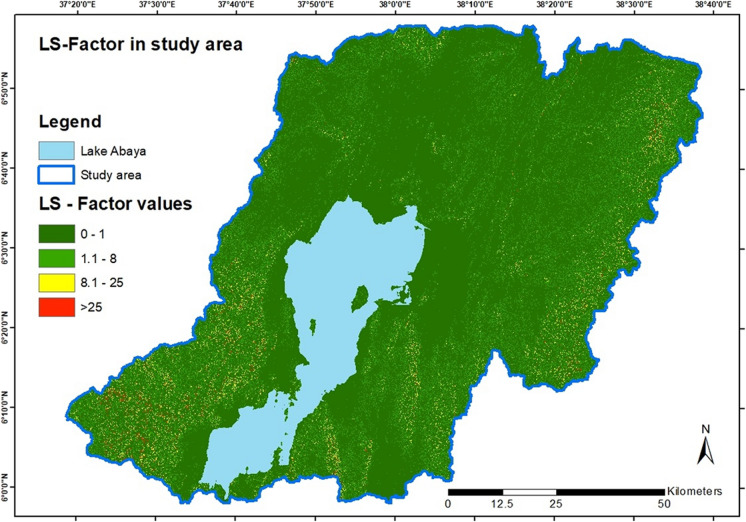
*LS*-factor in the study area

The results show the *LS*-factor clustering in a wide area in the east on the full length from north to south and in the southwest of the study area. Some smaller clusters are concentrated in the central south, east of Lake Abaya, and a cluster in the northern parts. The most extreme values > 25 are in the southwestern and with a smaller distribution in the east. Figure 4 shows the resulting layer for the area-wide *LS*-factor.

#### Land cover (C-factor)

**Fig. 5:**
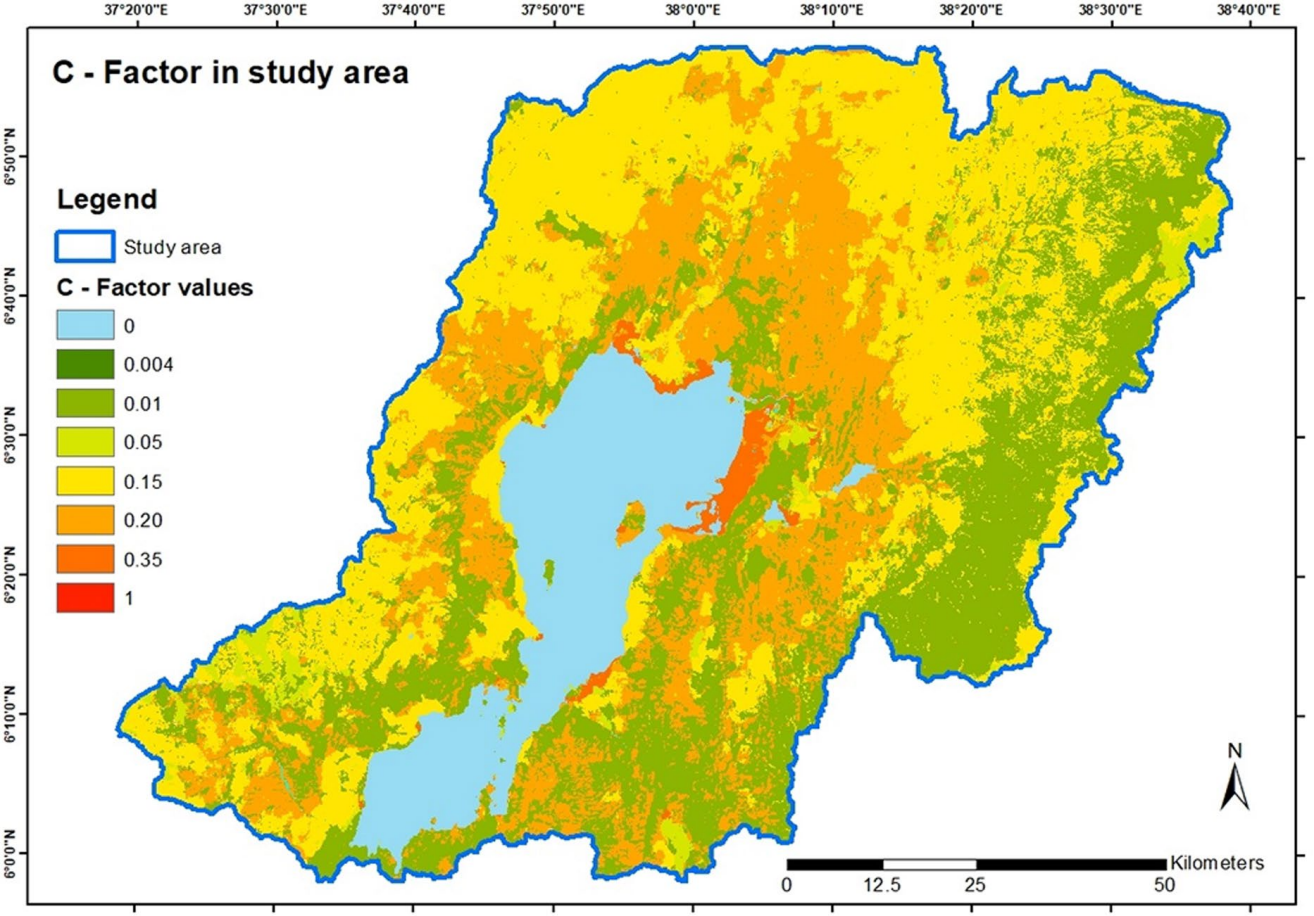
*C*-factor value distribution in the study area

The results show the areal expression of the *C*-factor from the *C* values associated with the land uses in the study area. The highest *C*-factor results from the *C* value of 1 for bare ground and 0.35 for flooded vegetation. It is particularly noticeable that the north and western parts of Lake Chamo are covered by flooded vegetation with a C value of 0.35. From there to the southwest and the eastern center, in the west to southwest and with smaller patches on the eastern border of the study area is covered by cropland, attaining 0.15 C value. The lowest *C*-factor is deduced from the *C* value of 0.01 for forests and 0.05 for grass, expressed widely in the east and with fewer total expressions around Lake Abaya. The intermediate areas are represented by an intermediate *C*-factor value of 0.2 for scrub/shrub land. Water areas are associated with a *C* value of 0. Figure 5 shows the resulting layer for the area-wide *C*-factor.

#### Soil conservation practice (P-factor)

**Fig. 6:**
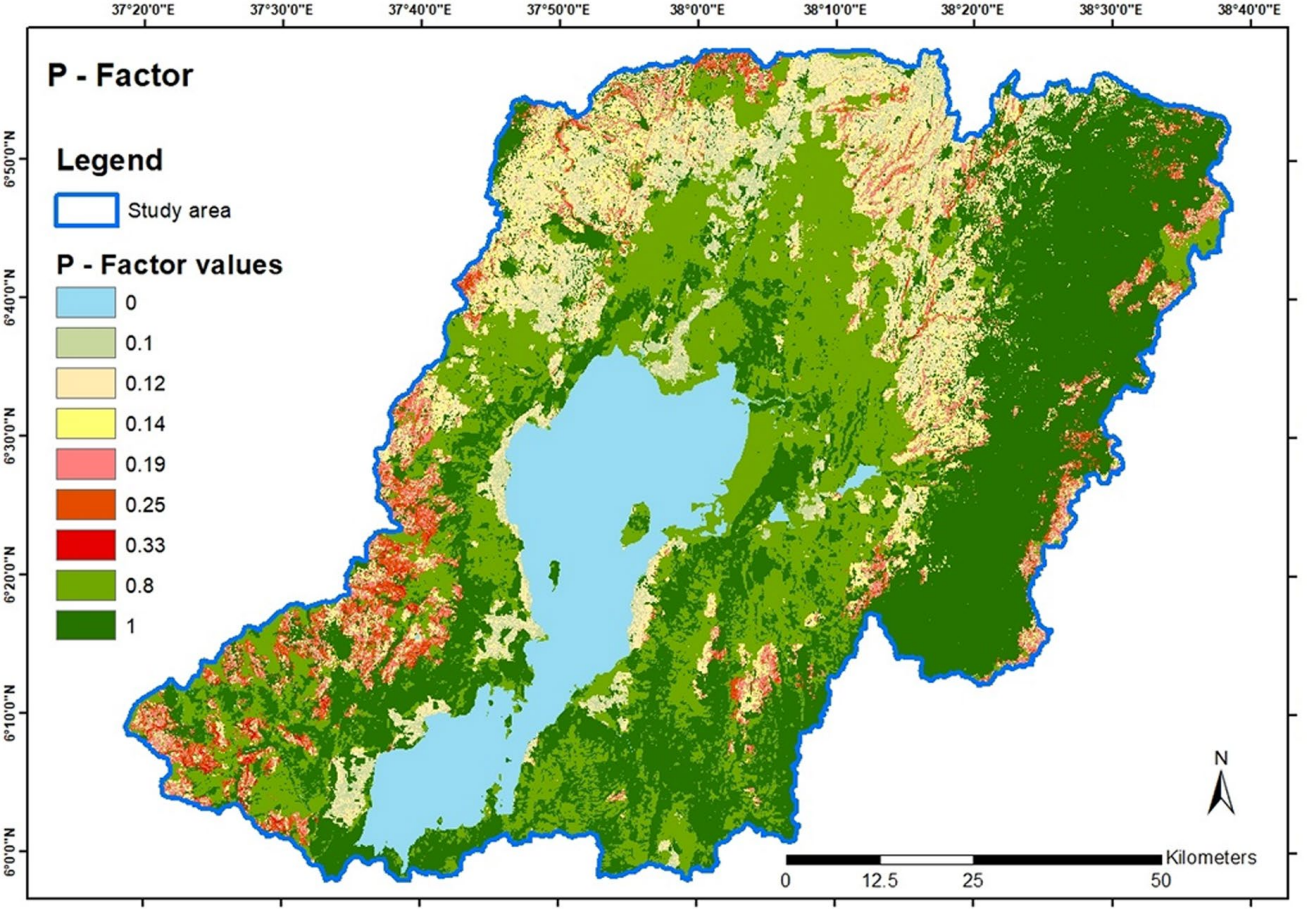
*P*-factor value distribution in the study area

The *P*-factor results show a wide variation of the *P* values, with a value of one associated with forests in the east, from north to south, and sparsely distributed around Lake Abaya intermixed with the *P* value 0.8 for moderately vegetated land. The *P* value zero associated with water is represented for Lake Abaya and some smaller water patches to the east of it. The *P* values for arable land show the most distribution in the north expanding to the southwest in the eastern center, mostly on low to moderate slopes with *P* values of 0.1 and 0.12. Another concentration of arable land is in the west but on much higher slopes represented by the higher *P* values of 0.19, 0.25, and even 0.33. Some arable land on mostly higher slopes is also situated at the most eastern study area, and some arable land is mostly on moderate slopes with a value of 0.12 on the shores around Lake Abaya. Figure 6 shows the resulting layer for the area-wide *P*-factor.

### Annual soil loss and erosion risk

Our results show that 73.49% of the catchment has a very low erosion risk. A total of 7.38% has a low, 5.94% has a medium, 7.32% has a high, and 5.872% has a very high erosion risk. The detailed erosion classes are presented in Table 4. Though the vast majority of the catchment falls under very low soil erosion risk, about 13% of the catchment has been identified as an erosion risk area above an average annual tolerable soil loss.

**Table 4:** Erosion risk classes with associated potential soil loss range

Erosion risk classes	Associated soil loss class to risk (t/ha/yr)	Area (ha)
Very low	0–0.5	617,808
Low	0.5–2	62,059
Medium	2–5	49,901
High	5–15	61,533
Very high	> 15	49,273
Total area (ha)		840,574

**Fig. 7:**
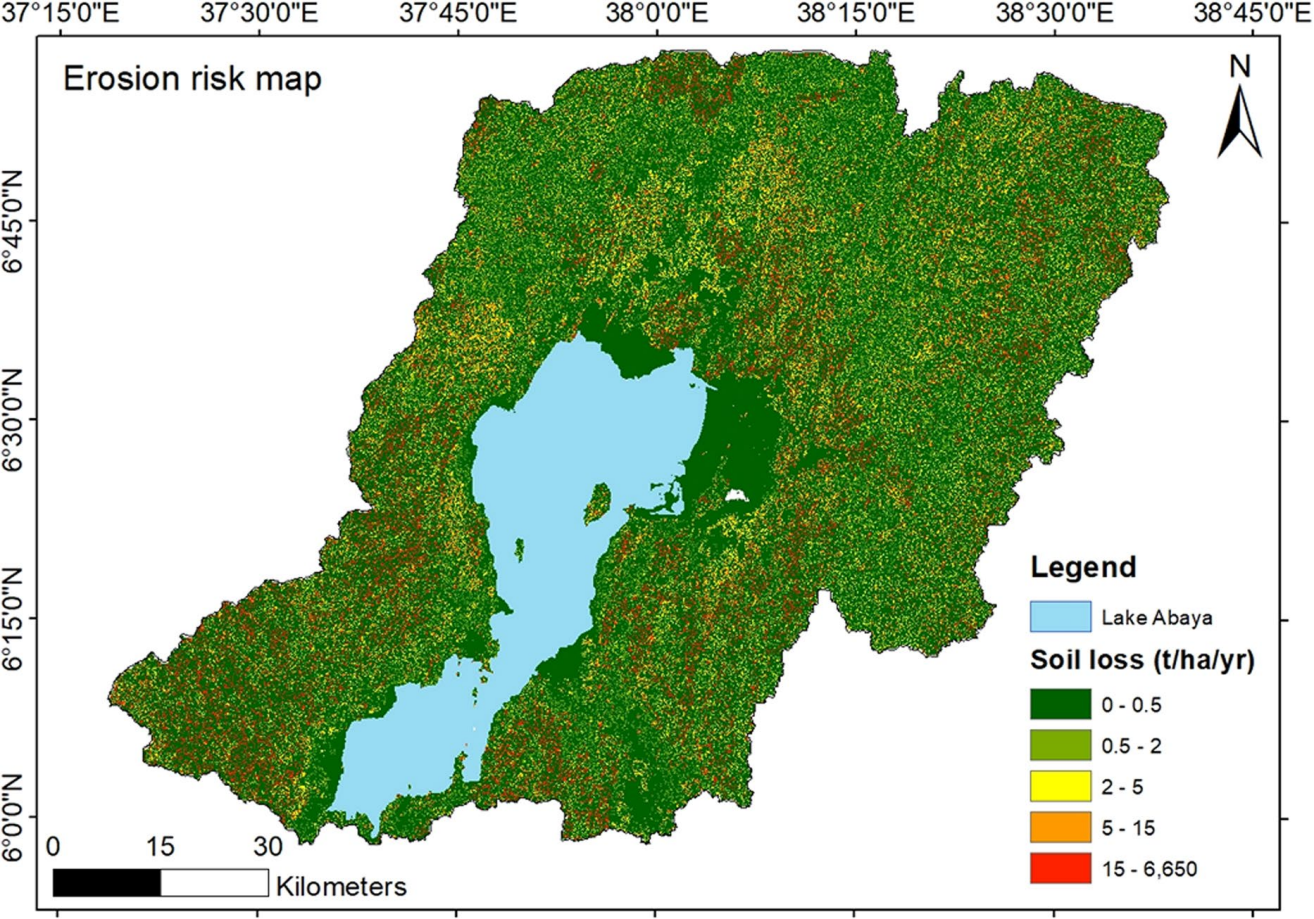
Erosion risk map of Abaya catchment

Hamessa and Baso Rivers from the west contributed to higher erosion risk, followed by the Bilate River in the north. Figure 7 illustrates the current erosion risk map of the study area.

**Fig. 8:**
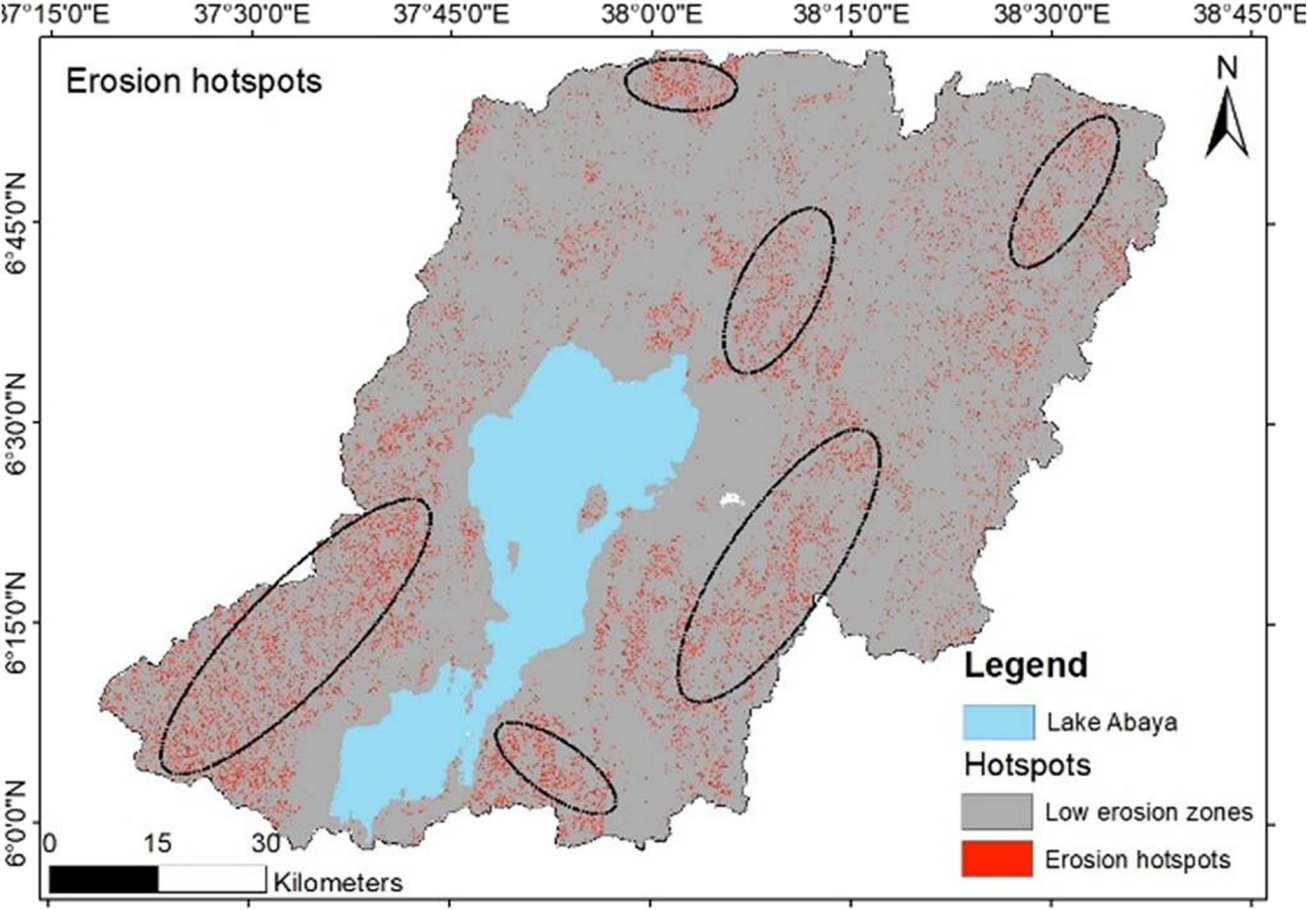
Erosion hotspot area before restoration

The result of the soil erosion risk depicted that the southwestern part of the Lake Abaya catchment has a higher annual soil loss risk than the other parts. Figure 8 shows the erosion hotspot areas before the implementation of scenario-based FLR implementation. Among the hotspot areas, the highlighted zones indicate areas with annual soil erosion above 15 t/ha/year.

### Impact of FLR on erosion reduction

**Fig. 9:**
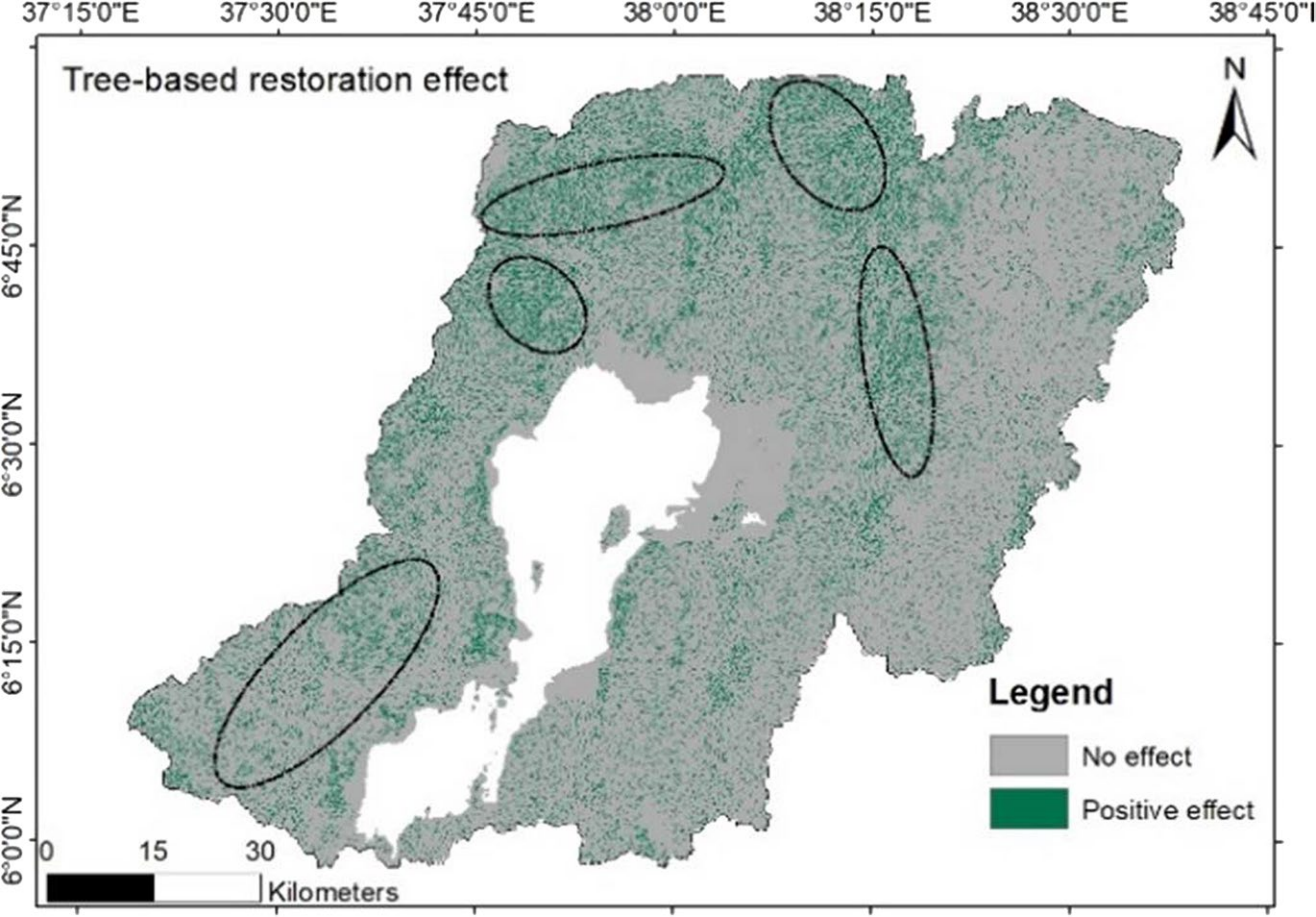
Expected positive impact on erosion hotspot areas after restoration

The simulation of the positive impact of area-wide restoration was estimated. The result depicted that restoring the landscape significantly reduced the land area under high soil erosion risk. The simulated FLR adjusts the *C*-factor from the five factors calculated in the RUSLE. The *C*-factor values of the bareland were re-established using the land cover of trees. Furthermore, the land cover under cropland situated on slopes between 30 and 60% has been assigned for agroforestry practice, whereby trees are integrated into the cropping landscape. Figure 9 illustrates how the proposed FLR strategies positively contribute to the reduction of the soil risk in the catchment.

Simulating the impact of FLR under the developed scenario resulted in a positive impact on about 14,000 ha of land in the catchment. The result from the scenario analysis is depicted in Fig. 9, where potential positive effects can be expected after implementing the area-wide restoration of bareland and the tree-based restoration of croplands.

**Fig. 10:**
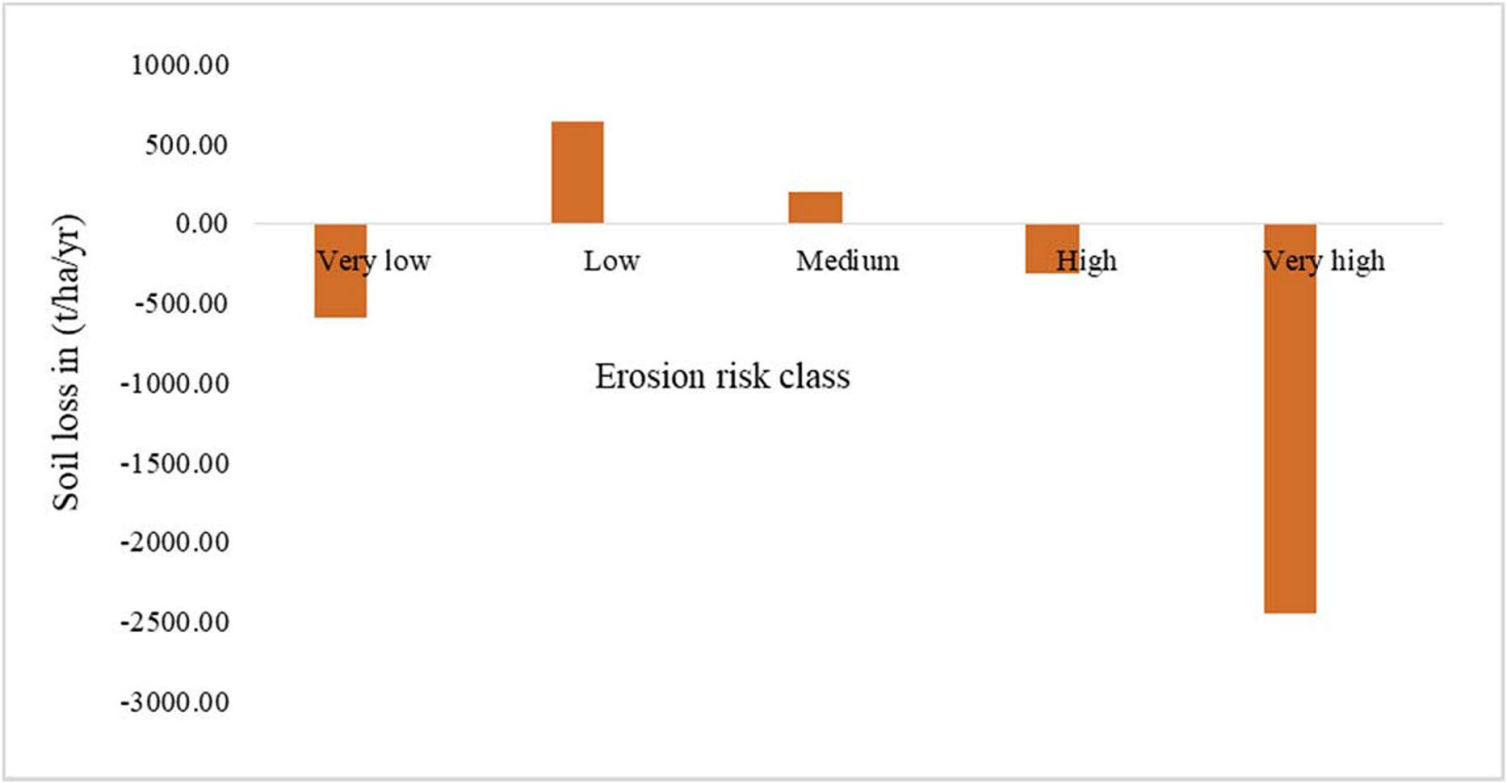
Changing soil erosion risk categories after simulated FLR scenario

The scenario analysis has resulted in shifting erosion class from very high to medium and low classes. From the category of high erosion risk areas, the scenario analysis of FLR implementation in the catchment implies an estimated reduction of soil loss to 3000 t/ha/year. By altering the *C*-factor in the calculation of the annual soil loss in the area, the study revealed a high possibility of lowering the soil loss in the area and moving down below annual tolerable soil loss from the catchment. Figure 10 illustrates a graphical presentation of erosion risk categories after the FLR scenario analysis integrated into the calculation of soil loss using RUSLE.

## Discussion

The results from the soil loss modeling revealed varied dynamics of soil loss and the effect of FLR activities on soil erosion in the Lake Abaya catchment. The spatial distribution of the individual RUSLE factors was consistent with the environmental conditions shaping them across the study region. For instance, the *R*-factor was consistent with the rainfall distribution in the study area. The results indicated that regions with high precipitation tend to have high *R*-factor values. Our results are consistent with other soil erosion estimation studies in the larger Rift Valley basin in the Eastern African region (Bekele & Gemi, 2021; Kogo et al., 2020). The influence of the *R*-factor is also notable from the overall erosion risk map of the study area. This coincides with Renard (1997), who showed that the *R*-factor has the largest influence on soil loss than other RUSLE factors. Similarly, Yang et al. (2013) indicated that the factor is highly correlated with soil loss and is an important factor in the RUSLE model.

The values for the *K*-factor also depicted variation across the study area. The high *K*-factor values spanned the north-south front of the study area. This is attributed to the region’s land cover and drainage system networks. The *K*-factor models the response of soil profile to detachment processes and subsequent transport by water runoff (Kogo et al., 2020). As such, the configuration of the drainage systems shaped by the topography and the bareness of the land surface are crucial modifications of erodibility in the catchment.

The *LS*-factor values distribution also influenced the soil loss estimation across the study area. Areas of steep slopes in the southwestern and southeastern parts of the study area showed an increased risk of soil erosion. According to Dananto et al. (2022), higher slopes accelerate sedimentation processes. This is brought about by the effects of increased surface runoff velocities and intensities. Thus, higher slopes combined with the reduced vegetation cover in the southwestern and southeastern zones are probable reasons for increased soil loss. Also, forested hilly slopes in the northeastern part of the study area depicted an increased risk of soil losses. This could be attributed to the combined effects of anthropogenic and topographical factors. A study by WoldeYohannes et al. (2018) shows that increasing anthropogenic influences caused by population growth, internal migration, and policy shifts have fuelled land fragmentation and negative environmental influences in the catchment.

The *C*-factor and *P*-factor in the study were derived mainly from the land-use characterizations drawn from the land use/land cover (LULC) map of the Lake Chamo catchment. In the present study, the land cover map defining the study area was used as a proxy for determining the spatial distribution of the factor values. However, other studies have defined the factors using proxies, such as the normalized difference vegetation index (NDVI) (Durigon et al., 2014; Kogo et al., 2020). The *C*-factor accounts for the land cover influence on the soil loss. The underlying rationale is that those areas that have extensive canopies and high tree densities are more likely to prevent soil loss and are therefore assigned low values. Thus, the factor is inversely related to the land cover intensity, with high vegetation cover recording a maximum value of one and low vegetation cover recording zero (Kogo et al., 2020). Effective land management practices at the watershed level play a critical role in reducing sediment flow to downstream water bodies (Kebede et al., 2021). Studies by Bekele and Gemi (2021) and Dananto et al. (2022) show that soil conservation practices such as terracing, contour farming, and strip cropping can potentially minimize soil loss. On the other hand, the *P*-factor accounts for conservation measures that reduce surface water runoff. Similarly, the values for the factor were interpreted from the LULC characterizations in the study region.

The Ethiopian Rural Land Proclamation clearly states that the land allocation for cropland should be limited to a certain slope category. However, farming along steep slopes is a common scenario in the study region which has contributed to high erosion risk. The results from the analysis show that the hotspot areas are associated with barelands and croplands and zones with steep slopes as indicated by the *LS*-factor.

Modeling of FLR potential measures enables the examination of their spatial influence on soil loss reduction. Thus, we assessed agroforestry and tree-based systems as potential practices in the study area. The practices are among the key FLR measures championed in highly degraded landscapes in Ethiopia (Kassa et al., 2022; Pistorius et al., 2017). Our results revealed the beneficial effects of restoration practices on soil erosion reduction. The results showed that restoring barelands and cropland under agroforestry has higher positive impacts than the sole restoration of barelands. The scenario analysis reveals a potential reduction of risk areas through the implementation of FLR in the area. This result is in tandem with Teng et al. (2019) who noted a significant soil erosion reduction under intensive forest restoration of areas within a range of 45 to 70% slope. Similarly, Wei et al. (2021) found that vegetation restoration greatly reduced soil erosion in China. Spatially, the FLR activities revealed huge effects in the northern part of the Lake Abaya catchment. The result is consistent with the physical characterization of the area, which shows that the northern part of the catchment is relatively more degraded than the other parts.

The eastern part of the Lake Abaya catchment and the area surrounding the lake revealed fewer effects of FLR activities on soil loss reduction. This is attributed to the flooded vegetation cover which is attributed to the topographical and vegetation combination effects. The eastern part of the catchment has a relatively higher forest cover than other parts of the study area. The vegetation cover management in this region is also optimal, and therefore, soil loss and land degradation are low in these zones. Agroforestry activities, already in practice in the study area, need to be intensified and expanded to reduce soil loss (Zekarias et al., 2021).

In terms of severity of soil loss, the study showed that most parts of the study area have low to medium severity of soil loss. The average annual tolerable soil loss in Ethiopia is about 10 t/ha/year (Girmay et al., 2020; Taddese, 2001). The area which exceeded the average annual tolerable soil loss is 110,000 ha which still needs to be considered for possible reversal mechanisms of the erosion. These areas should be prioritized for FLR intensification, especially in light of the growing population and the high demand for food and energy in the study area. Ethiopia is one of the most highly populated nations in sub-Saharan Africa and more demand for livelihood services will put environmental resources under pressure in the future (Haile, 2004).

## Conclusion

In assessing the impacts of FLR toward combating soil erosion, our research revealed that deforestation of the upper catchment coupled with the rugged terrain of the landscape resulted in a high erosion risk in the Abaya catchment. The northern and southwestern parts of the catchment are highly erosion risk areas which are highly linked with the land-use and terrain characteristics. Our study revealed that FLR activities, especially tree-based systems, hold an enormous potential for reversing erosion effects in the study region. Furthermore, the study highlighted key hotspot zones where FLR projects need to be intensified to abate further erosion and improve the state of the landscape. Therefore, we argue that to increase the productivity of the land and the ecosystem service provision, the implementation of area-wide restoration through different FLR options is required. Particularly, to minimize the erosion risk in the catchment, tree-based agroforestry practice on the farmlands is a convenient measure. Moreover, areas under medium risk severity also need to be conserved from soil losses, as the phenomenon is a continuous process that can cause devastating effects over the years.

As policies and directives related to land use are customary in order to enhance the ecological functionality of the landscape and the livelihood of the community, abiding by the regulation is a way forward in reducing environmental issues such as soil erosion risk. Thus, FLR activities hold great potential for minimizing soil loss and contributing to supporting functioning and providing ecosystem services. Moreover, integrating land management through implementing diverse soil and water conservation activities together with the land cover factor can potentially increase the contribution of FLR in reducing soil loss. Hence, local communities and projects working on FLR implementation in the area can target the two factors especially on the land area with steep slopes. Implementation of FLR prioritization with soil loss from the catchment alone but other ecological and socio-economic aspects must also be included on an equal footing. Therefore, research on the integration of socio-economic factors of the local community living in the catchment in relation to soil erosion risk is important to tackle the problem from both ecological and socio-economic perspectives.

### Supplementary information

ESM 1 (DOCX 327 kb)

## Data Availability

Data and materials are available upon request.
