# Supplementary material for: Impact of forest landscape restoration in combating soil erosion in the Lake Abaya catchment, Southern Ethiopia
Source: Environ Monit Assess. 2024 Feb 2;196(3):228. doi: 10.1007/s10661-024-12378-8 (PMC10837221; doi:10.1007/s10661-024-12378-8)
Supplement: Supplementary file 1 — (DOCX 327 kb) [file 10661_2024_12378_MOESM1_ESM.docx]

**Supplementary File**


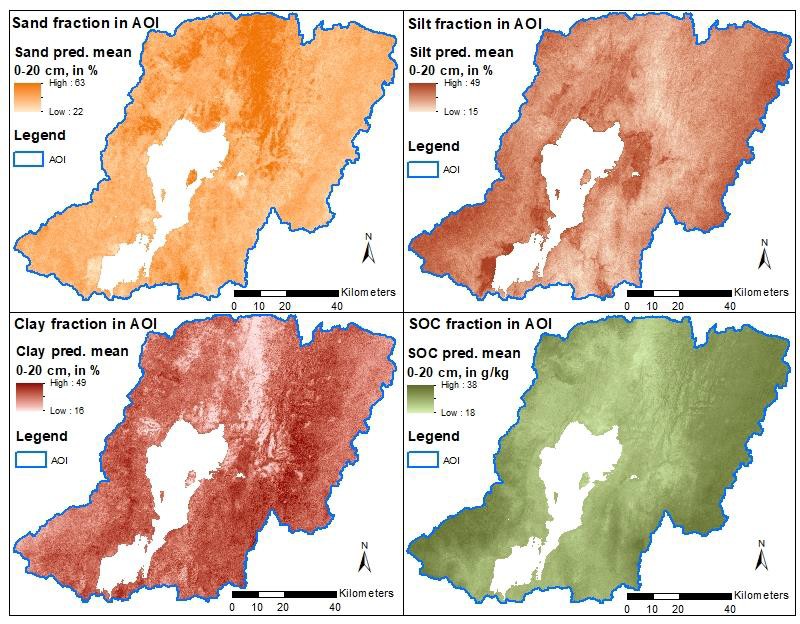


Fig. S1 Soil maps used to compute soil erodibility (K-factor)


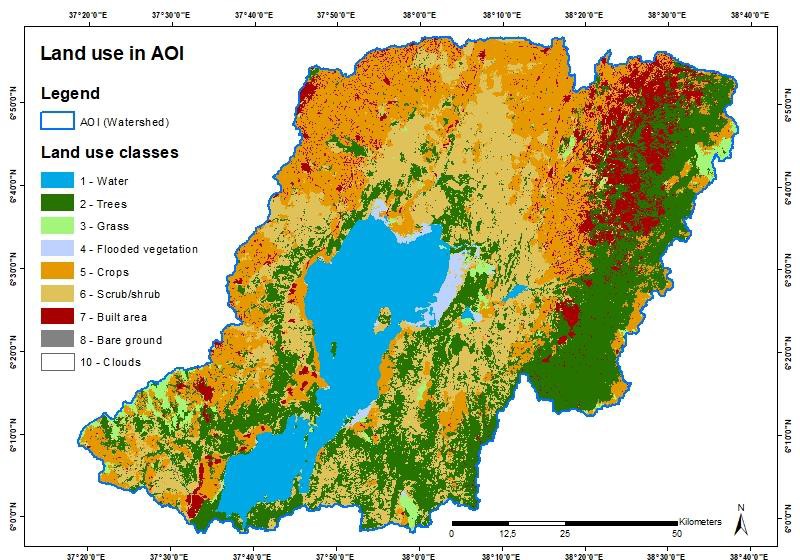


**Fig. S2** Land use land cover map from ESRI used to compute with the landcover (C values)
